# When infection hurts: golden rules for managing pediatric skin and soft tissue infections

**DOI:** 10.1186/s13052-025-01994-w

**Published:** 2025-06-17

**Authors:** Giangiacomo Nicolini, Valentina Frasca Polara, Matteo Calvi, Marc García-Lorenzo, Miriam Massese, Gregorio Paolo Milani, Roberta Parrino, Enrico Vidal, Claudia Colomba

**Affiliations:** 1 Pediatric Care Unit, San Martino General Hospital, Belluno, 32100 Italy; 2 Division of Paediatric Infectious Disease,“G. Di Cristina”Hospital, ARNAS Civico Di Cristina Benfratelli, Palermo, 90100 Italy; 3 Pediatric Emergency Department, ASST Papa Giovanni XXIII Hospital, Bergamo, 24127 Italy; 4 Pediatric Infectious Disease Unit, Department of Paediatrics, Luigi Sacco Hospital, University of Milan, Milan, 20157 Italy; 5 Department of Pediatrics, Fondazione Policlinico Universitario A. Gemelli IRCCS, Rome, 00168 Italy; 6 Pediatric Unit, Fondazione IRCCS Ca’ Granda Ospedale Maggiore Policlinico, Milan, 20122 Italy; 7 Department of Clinical Sciences and Community Health, Università Degli Studi Di Milano, Milan, 20122 Italy; 8 Pediatric Nephrology Unit, Department of Women’s and Children’s Health, University of Padua, Padua, 35128 Italy; 9 Department of Medicine (DMED), University of Udine, Udine, 33100 Italy; 10 Department of Health Promotion, Mother and Child Care, Internal Medicine and Medical Specialties “G. D’Alessandro”, University of Palermo, Palermo, 90100 Italy

**Keywords:** Skin and soft tissue infections, Pediatric care, Antimicrobial stewardship, Empirical therapy, Pain management, Fever control

## Abstract

Skin and Soft Tissue Infections (SSTIs) are common in pediatric patients, accounting for nearly 25% of clinical visits. These infections can range from mild to life-threatening and include a severe subset known as Acute Bacterial Skin and Skin Structure Infections (ABSSSI). Prompt diagnosis and appropriate antibiotic use are crucial for optimizing patient outcomes while minimizing adverse effects and antimicrobial resistance. However, empirical treatment often becomes necessary due to the lack of culture specimens, making local epidemiology and clinical presentation key factors in treatment decisions. This expert opinion paper aims to outline the “*golden rules*” for the management of SSTIs in children, focusing on achieving microbiological clearance, clinical improvement, and effective control of symptoms, such as fever and pain, which significantly impact the child's well-being. These emphasize the principles of antimicrobial stewardship, recommending early diagnosis with appropriate laboratory tests, rational empiric therapy, and prompt switch to targeted therapy based on microbiological findings, as well as proper fever and pain management. The paper also highlights the importance of a multidisciplinary approach for complex cases, optimal dosing, and effective communication with patients' families to improve treatment compliance. Furthermore, antibiotic therapy should be selected to reduce hospital stay and facilitate home-based continuity of care, while follow-up and strengthening of the hospital-territory network are critical for continuity of care after discharge. These recommendations aim to optimize the management of pediatric SSTIs by ensuring comprehensive care from initial diagnosis to post-discharge follow-up, promoting the rational use of antibiotics, and ultimately improving clinical outcomes and quality of life for children and their families.

## Introduction

Skin and Soft Tissue Infections (SSTIs) are among the most common bacterial infections in children, accounting for nearly 25% of clinical encounters and leading to 3 million healthcare visits/year [1]. SSTIs are clinical entities of variable presentation, ranging from mild and local to life-threatening systemic infections [2]. SSTIs can be classified according to several criteria: severity (mild, moderate, or severe), skin involvement (uncomplicated infections, typically superficial, and complicated infections, usually with deeper tissue involvement), rate of progression (acute or chronic wound infections), presence of pus (purulent or non-purulent infections) and tissue necrosis (necrotizing or non-necrotizing infections) [3, 4]. A new disease category called Acute Bacterial Skin and Skin Structure Infections (ABSSSI) has been proposed to better define a severe subgroup of SSTI, including cellulitis, erysipelas, major skin abscesses, and wound infections with a minimum lesion area of 75 cm^2^ [5].

Prompt recognition and the timely and appropriate use of antibiotics are critical to optimize patient outcomes and minimize the occurrence of adverse drug effects and the emergence of antimicrobial resistance. As specimens for culture are often not obtained for many SSTIs, treatment tends to be empirical, based on clinical presentation and local microbial epidemiology, with *Staphylococcus aureus*, including methicillin-resistant (MRSA) strains, and *Streptococcus pyogenes* as the most common causes of community-onset SSTIs [6, 7].

Inflammation plays a central role in the immune response to bacterial skin infections and serves as a defense mechanism against pathogens. However, the inflammatory process triggered by infection often results in signs and symptoms, such as fever and pain, which can have a significant impact on a child's well-being [8, 9]. For this reason, drugs with analgesic and antipyretic properties have been commonly used in infections as supportive treatments to relieve associated symptoms, alongside empiric treatments selected on the basis of the diagnostic process.

Recent epidemiological data confirm the significant burden of SSTIs in pediatric patients. According to the EUCLIDS study, which analyzed over 2,800 children hospitalized for severe infections across Europe, SSTIs accounted for 8.7% of all focal infections, with *Staphylococcus aureus* and *Streptococcus pyogenes* as the main pathogens [10]. Furthermore, impetigo, a highly contagious superficial SSTI, affects more than 162 million children worldwide at any given time, making it one of the most prevalent pediatric infections globally [11]. These data are consistent with previous findings that SSTIs account for approximately 10% of all hospital admissions due to infections [11]. Additionally, an increase in emergency department visits and hospitalizations related to SSTIs has been reported in young children over recent decades [2]. Taken together, these findings highlight the widespread prevalence and clinical impact of SSTIs in children, reinforcing the need for structured management approaches based on multidisciplinary collaboration [12].

Given the high prevalence and diverse clinical manifestations of SSTIs in pediatric patients, it is essential to establish effective management strategies that not only ensure timely diagnosis and appropriate treatment, but also improve patient comfort through symptom control.

Therefore, the aim of this expert opinion paper is to analyze and outline the best practices in the management of SSTIs in pediatric setting, with a focus on achieving microbiological clearance and clinical improvement, while also addressing fever and pain management.

## Methods

Following an in-depth review of the current literature using the Pubmed/MEDLINE database, nine Italian experts in pediatrics and infectious diseases met on July 2, 2024, in Milan, Italy, to discuss and develop “Golden Rules” for helping all specialists involved in the management of SSTIs in pediatric patients, also considering the importance of fever and related pain control. These recommendations are based on a comprehensive literature review combined with the practical insights and clinical experience of experts to ensure that they are evidence-based and applicable in real-world settings. The goal is to outline general principles that integrate and improve clinical practices, adapting them to local contexts, antibiotic resistance patterns, and epidemiological data.

## Management of pediatric infections in the presence of fever and pain

### Principles of antimicrobial stewardship and their application in pediatric infections

In 2021, antibiotics accounted for more than 10% of the total drug expense of Italy's National Health System, highlighting their crucial role in treating bacterial infections but also raising concerns about their appropriate use [13]. Inappropriate use of antibiotics, influenced by cultural attitudes, has led to a significant increase in adverse effects, the most critical of which is antimicrobial resistance (AMR). Additionally, *Clostridioides difficile*-associated diarrhea, resulting from dysbiosis due to antibiotic pressure, should not be underestimated. The overuse of antibiotics in pediatric patients is also associated with higher rates of asthma, allergies, and childhood obesity [14–17].

Antimicrobial stewardship programs are a set of interventions aimed at improving the appropriate use of antibiotics, with the goal of obtaining the best clinical outcome through minimizing adverse events, limiting the emergence of drug resistance, and therefore reducing costs [18, 19]. Every AMS-program must be based on prompt diagnostic antimicrobial stewardship aimed at identifying the causative pathogen, allowing for a shift from empirical to targeted antibiotic therapy. For this reason, it is crucial to perform culture tests before initiating antibiotic treatment. These programs are more commonly implemented in adult settings but establishing them for pediatric patients is challenging due to their smaller numbers and different care settings [20]. Developing a pediatric antimicrobial stewardship program requires the formation of a multidisciplinary team to create guidelines, monitor prescriptions, and ensure administrative and financial support. The goal is to educate pediatricians and ensure that stewardship principles are effectively integrated into hospital and community clinical activity to improve good practice in antibiotic prescribing and ultimately reduce the burden of antimicrobial resistance and associated healthcare costs.

### Therapeutic options with anti-MRSA activity for the treatment of SSTIs/ABSSSIs in children

The clinical management of SSTI/ABSSSI is multifaceted and usually involves antibiotic therapy. The presence of comorbidities, the need for dose adjustments, and potential drug interactions further complicate drug selection and patient management [3, 21].

When selecting an effective antibiotic for the treatment of SSTIs/ABSSSIs, the following factors should be considered: (i) the spectrum of activity, especially for empiric therapy; (ii) the safety profile; (iii) pharmacokinetic properties; (iv) its suitability for outpatient treatment; (v) the feasibility of switching from intravenous to oral administration for early discharge; and (vi) patient adherence to the treatment regimen [12, 22].

Currently, several oral and parenteral therapeutic options with activity against MRSA are available for the management of SSTIs/ABSSSIs in children. The main characteristics of the most frequently used drugs in children are briefly summarized in Table 1.

**Table 1:** Antibiotics with anti-MRSA activity for the treatment of SSTI/ABSSSI in children

**Antibiotic**	**Class**	**Antimicrobial activity**	**Route of administration and Infusion Time**	**Dosing Schedule**	**Adverse Effects**
Vancomycin	Glycopeptide	Gram-positives	Intravenous 60 min	Neonates: 10 to 15 mg/kg every 12 h in the first week of life and every 8 h thereafter until one month of age. The dosage is calculated according to post-menstrual age Infants and children from one month to 12 years: 10 to 15 mg/kg every 6 h for 7–14 days Children ≥ 12 years: 15 to 20 mg/kg every 8 to 12 h for 7–14 days	• Renal toxicity • Flushing syndrome • Need of TDM • Multiple daily intravenous administrations
Teicoplanin	Glycopeptide	Gram-positives	Intravenous or intramuscular 30 min	Neonates and infants up to 2 months: 16 mg/kg on the first day followed by a maintenance dose of 8 mg/kg/day Infants and children from 2 months to 12 years: 10 mg/kg every 12 h repeated 3 times followed by 6–10 mg/kg/day Length of treatment varies depending on individual response to treatment	• Renal toxicity (reduced compared to vancomycin) • Flushing syndrome (reduced compared to vancomycin) • Repeated intravenous administrations
Tedizolid	Oxazolidinone	Gram-positive, including linezolid-resistant strains	Intravenous and oral NA	Children ≥ 12 years: 200 mg/day for 6 days	• Not approved in children below 12 years of age • Drug interactions • Gastrointestinal disorder and myelotoxicity
Daptomycin	Lipopeptide	Gram-positive, including vancomycin-resistant strains	Intravenous 30 to 60 min depending on age group	Children aged 12 to 17 years: 5 mg/kg/day up to 14 days Children aged 7 to 11 years: 7 mg/kg/day up to 14 days Children aged 2 to 6 years: 9 mg/kg/day up to 14 days Children aged 1 to 2 years: 10 mg/kg/day up to 14 days	• Myopathy • Daily intravenous administration
Ceftaroline	Beta-lactam (fifth-generation cephalosporin)	Gram-positive and Gram-negative	Intravenous 5 to 60 min depending on age group	Adolescent aged 12 to 18 years and bodyweight > 33 kg: 600 mg q12 h Adolescents aged 12 to 18 years and bodyweight < 33 kg: 12 mg/kg to a maximum of 400 mg q8 h Children aged 2 to 12 years: 12 mg/kg to a maximum of 400 mg q8 h Infantes from 2 months to 2 years: 8 mg/kg q8 h Neonates from birth to 2 months: 6 mg/kg q8 h	• Poor activity against *Enterococcus* • Multiple daily doses
Dalbavancin	Lipoglycopeptide	Gram-positive	Intravenous 30 min	Children and adolescents aged from 6 years to less than 18 years: single dose of 18 mg/kg (maximum 1,500 mg) Children aged from 3 months to less than 6 years: single dose of 22.5 mg/kg (maximum 1,500 mg)	

Despite the availability of many treatment options, optimal management of SSTIs remains challenging. These drugs often require multiple daily doses and are associated with gastrointestinal side effects, resulting in poor adherence and suboptimal clinical outcomes. In addition, overuse and prolonged duration of antibiotic treatments may contribute to the development of drug resistance.

## Golden rules for managing pediatric SSTIs in the presence of pain and fever

The ten “golden rules” for the effective management of pediatric SSTIs in the presence of pain and fever are presented in Table 2.

**Table 2:** Golden rules for pediatric SSTI management in the presence of pain and fever

N	Key areas of pediatric infection management	Recommendations	Rationale
1	VACCINATION HISTORY AND IDENTIFICATION OF RISK FACTORS	Investigate the child's immunization status and the existence of any possible risk factor for bacterial infections (recent hospitalizations, recent antibiotic therapy, steroid therapy, chronic illness, recent travel abroad, hygienic-sanitary conditions, health status of the family, colonization status)	Systematic collection of relevant anamnestic data is critical to guide treatment choices and identify early patients at risk of serious bacterial infections or usually covered by vaccination, enabling more effective preventive strategies and individualized interventions
2	EARLY DIAGNOSIS	Evaluate which patients need blood tests, culture and laboratory tests before starting empiric antibiotic therapy	Careful and reasoned evaluation of diagnostic tests to be performed before initiating empiric antibiotic therapy increases the sensitivity and specificity of these tests, improving the accuracy of diagnosis and allowing therapy to be tailored appropriately
3	RATIONAL EMPIRICAL THERAPY	Identify the correct empirical antimicrobial therapy based on local epidemiology, site of infection, possible cause of infection, and host characteristics	Personalization of empirical therapy, based on local epidemiology and individual patient specifics, can improve the effectiveness of initial treatment and reduce the likelihood of treatment failure
4	TARGETED THERAPY	Modify antibiotic therapy promptly according to the results of microbiological tests	Timely adjustment of antibiotic therapy based on diagnostic findings allows optimization of targeted therapy, reducing bacterial resistance selection and improving clinical outcomes
5	FEVER AND PAIN MANAGEMENT IN RELATION TO CLINICAL PROFILE	Focus the history on the possible use of antipyretics/analgesics for fever/pain management, specifically asking if paracetamol or NSAIDs have already been used before and then consider any concomitant factors that may increase drug toxicity in order to set up appropriate strategies for fever and pain management during infection	Careful evaluation of factors that may increase the toxicity of antipyretic and pain-relieving therapy makes it possible to optimize the efficacy of symptomatic treatment and minimize the risk of adverse events, while also reducing the possibility of worsening potentially invasive infections
6	MULTIDISCIPLINARY INTERVENTIONS	Manage complex and/or complicated cases as a multidisciplinary team for more comprehensive and coordinated management of the pediatric patient	The multidisciplinary approach to complex and/or complicated cases ensures improved clinical outcomes through more comprehensive and coordinated management of the pediatric patient
7	BETTER ASSESSMENT OF APPROPRIATE DOSAGES AND ADMINISTATION INTERVALS	Carefully assess appropriate dosages during hospitalization and provide appropriate health education to parents/caregivers of patients regarding dosages and hourly intervals of administration of home-prescribed medications to improve therapeutic compliance	Proper education of the pediatric patient's family improves adherence to prescribed therapy and reduces the risk of medication errors, increasing the effectiveness and safety of care
8	APPROPRIATE COMMUNICATION WITH THE PATIENT'S FAMILY	Improve communication (active/passive) between doctor-caregiver-patient, both in terms of quality and time spent on it	Effective and clear communication with the pediatric patient's family is critical to establishing a physician-caregiver-patient relationship based on trust, thereby improving compliance, understanding of the care plan, and collaboration, which are essential elements for therapeutic success
9	MANAGMENT OF ANTIBIOTIC THERAPY	Make the best treatment choices based on comprehensive patient care, by choosing an antibiotic that reduces hospital stay, provides possible continuity for home care, and whose duration complies with the latest recommendations and guidelines	A holistic approach to antibiotic therapy reduces hospital infections, bacterial resistance, and the direct and indirect costs associated with prolonged hospitalizations, improving the quality of care overall
10	POST-DISCHARGE FOLLOW-UP	Strengthen the hospital-territory network, including cooperation with family pediatricians, to ensure continuity of care post-discharge	An integrated network of structured care and follow-up reduces the risk of re-hospitalizations and allows for better collaboration and sharing of therapy choices, enabling better quality of life for patients, greater efficiency of the health care system, and increased therapeutic adherence by parents of young patients

## Recommendation No. 1: Vaccination history and identification of risk factors

### Investigate the child's immunization status and the existence of any possible risk factor for bacterial infections (recent hospitalizations, recent antibiotic therapy, steroid therapy, chronic illness, recent travel abroad, hygienic-sanitary conditions, health status of the family, colonization status)

Systematic collection of relevant medical history data is essential for several reasons. First, it helps to guide treatment decisions accurately by providing a detailed overview of the child's health [23]. In addition, early identification of risk factors allows for the prompt detection of children who may be at higher risk for severe bacterial infections [24]. The presence of atopic dermatitis, skin lesions and trauma, insect bites and animal scratches, varicella, congenital immunodeficiencies, chemotherapy, poor hygiene, and MRSA colonization increase the susceptibility for SSTIs. Risk factors for MRSA colonization in children include age younger than 6 months or between 8 and 13 years, male sex, the presence of a family member previously hospitalized or working in a healthcare facility, high number of siblings, frequent visits to healthcare facilities or previous hospitalization, history of a permanent catheter or other medical device, chronic skin conditions, and chronic sinusitis [25–27].

Recently hospitalized children may have been exposed to nosocomial infections, and those who have recently undergone antibiotic or steroid treatment may have a compromised immune system. Chronic illnesses may also increase a child's susceptibility to infection, and recent international travel may result in exposure to uncommon pathogens. Assessing the child's living conditions, including hygiene practices and sanitary standards, is crucial to identify the possible environmental factors contributing to SSTI development. Additionally, understanding the health status of family members can help identify potential sources of infection and transmission within the household [25].

## Recommendation No. 2: Early diagnosis

### Evaluate which patients need blood tests, culture and laboratory tests before starting empiric antibiotic therapy.

The diagnosis of a SSTI/ABSSSI is generally based on clinical evaluation rather than microbiological results. This approach relies on the physician's evaluation of the patient's symptoms, physical examination findings and history to identify the infection [12].

However, microbiological diagnosis should be obtained in case of severe infections to guide more precise treatment, hospitalized patients to adjust antimicrobial therapy and prevent complications, and in case of poor responses to initial treatment to identify potential resistance or alternative pathogens and adjust the treatment regimen [3]. Effective selection and scheduling of diagnostic tests increase the chances of identifying the correct pathogen and improving treatment plans [28].

For common and simple SSTIs like cellulitis or small abscess, culture is usually not necessary, whereas in case of complicated SSTIs, samples should be collected and sent quickly to the microbiology laboratory with detailed information [29]. In case of abscess lesions, the first and most important interventions are incision and drainage, which allow for the collection of culture material, and aid antibiotic efficacy. Further, in case of persistent fever or signs of sepsis, blood culture is warranted. For these reasons, consultations with microbiologists are crucial at this phase.

Accurate diagnostic testing allows clinicians to tailor antibiotic therapy to target specific pathogens, optimizing treatment outcomes and helping to combat antibiotic resistance by reducing the use of broad-spectrum antibiotics.

## Recommendation No. 3: RATIONAL EMPIRICAL THERAPY

### Identify the correct empirical antimicrobial therapy based on local epidemiology, site of infection, possible cause of infection, and host characteristics.

In general, for SSTIs the choice of initial empiric therapy should be based on the pathogens most likely to be involved in the specific infection and local antimicrobial susceptibility patterns, pending culture results. The clinician should be guided by the patient's clinical presentation, along with the medical history and physical examination to identify probable pathogens and guide the selection of appropriate empirical treatment.

Although broad-spectrum regimens may be the most effective, their use must be carefully considered because of their higher costs and greater risk of promoting drug resistance. Conversely, narrow-spectrum antibiotics may carry a higher risk of treatment failure. When initiating empiric antibiotic therapy for SSTIs, clinicians try to balance these factors, with the goal of minimizing the risk of treatment failure while managing healthcare costs and mitigating the potential for bacterial resistance development [28].

Since bacterial skin infections are primarily caused by *S. aureus*, *S. pyogenes*, and other beta-hemolytic Streptococci, coverage against Gram-positive bacteria is commonly used as an empiric therapy for these conditions. Before the spread of MRSA as a cause of skin infections, antibiotic therapy was relatively simple, using an empiric approach with a β-lactam as the preferred therapy [30]. Since empirical antibiotic therapies are often ineffective against MRSA, most guidelines recommend the use of empirical treatments targeted at locally prevalent MRSA strains or reserved for cases of clinical instability [31, 32].

Inadequate initial antibiotic treatment for bacterial skin infections has been associated with treatment failure, higher rates of recurrence and readmission, longer hospital stays, higher healthcare costs and increased mortality [33, 34]. To ensure adequate empirical coverage, it is critical to understand local microbial epidemiology through routinely prepared microbiological maps and to accurately assess risk factors for skin infections sustained by MRSA. In daily clinical practice, this can be complex because of the diverse and numerous characteristics of high-risk patients, including disease- and host-related factors. Therefore, implementing an antimicrobial stewardship program can help reduce the duration of therapy and hospital stay, thereby optimizing patient care [35].

## Recommendation No. 4: Targeted therapy

### Modify antibiotic therapy promptly according to the results of microbiological tests.

The efficacy of empiric antibiotic therapy should be evaluated 48–72 h after treatment initiation by assessing culture results, potential resistance patterns of the causative pathogen, and the patient's clinical status, such as fever and inflammatory markers, to determine whether the antibiotic regimen should be modified [33].

The re-evaluation of antibiotic treatment is mandatory as soon as an antibiogram becomes available. While it is necessary to assess the susceptibility of the isolated pathogen to the currently administered antibiotic(s), it is equally important to narrow the spectrum of antibiotic used to more precisely target the specific microorganism. Thus, when microbiological data become available, antibiotic treatment should be replaced, ideally by a molecule only active against the isolated microorganism [28].

Limiting the use of broad-spectrum antibiotics is critical to avoid disrupting the patient's natural microbiota and selecting resistant strains, which can lead to serious and difficult-to-treat infections.

Therefore, therapeutic de-escalation should involve re-evaluation and possible modification of the antibiotic regimen based on clinical improvements and new microbiological data, aiming to use the least number of antibiotics, preferably a single agent with the narrowest spectrum, and considering the switch from parenteral to oral therapy, if appropriate [36]. In this process, the infectious disease specialist has a crucial role.

## Recommendation No. 5: Fever and pain management in relation to clinical profile

### Focus the history on the possible use of antipyretics/analgesics for fever/pain management, specifically asking if paracetamol or NSAIDs have already been used before and then consider any concomitant factors that may increase drug toxicity in order to set up appropriate strategies for fever and pain management during infection

Although fever should be treated only when there is concomitant discomfort, recent data suggest that defining discomfort can be difficult in clinical practice [37]. Fever and pain are challenging symptoms common in infants and children [38, 39]. Fever management tends to be characterized by overtreatment due to parental anxiety and fever phobia, meanwhile pain is undertreated, resulting in untimely and inadequate analgesia [40–42].

As in most cases fever is a physiological defense mechanism, antipyretics should be given only to relieve the child's discomfort and not to treat the fever itself. Therefore, they should not be administrated on the basis of a specific body temperature, but according to the child's clinical presentation; indeed, fever may be associated with various clinical symptoms, such as irritability, alterations in sleep–wake rhythm, feeding difficulties, headache, chills, and myalgias [43, 44]. In contrast, pain is often undertreated but should always receive adequate attention [45].

There is no superiority difference between paracetamol and ibuprofen in terms of effectiveness in treating fever and pain, but the safety profiles of the two drugs differ in some contexts.

Therefore, in light of precision medicine, it is possible to identify the categories of patients for whom the two drugs are most appropriate [46–48]. Inappropriate use of antipyretic drugs for fever or mild to moderate acute pain in children and adolescents may lead to increased risk of toxicity [49].

Paracetamol and ibuprofen are the only antipyretic drugs recommended for children, but their combined or alternate use is not recommended, considering risks and benefits [46, 47]. According to a recent systematic review, although combined or alternating therapy reduces temperature more effectively than a single antipyretic, the benefit on discomfort in children is not clinically significant [50]. This evidence cannot support the combined or alternating use of the two drugs compared to monotherapies, in agreement with most international recommendations [46, 51].

Both ibuprofen and paracetamol are generally safe for use in children when administered at therapeutic doses, with characteristic differences in the incidence of adverse events between the two drugs [52–55]. Possible concomitant factors that may increase the safety risk of antipyretic drugs should be considered [47].

Ibuprofen should be used with caution in cases of dehydration due to the increased risk of serious renal impairment, even when dosed correctly [56–60]. It is not recommended in children with active varicella due to the potential increased risk of skin and soft tissue superinfections and invasive streptococcal infections (e.g., empyema) and it is contraindicated in children treated with ACE inhibitors or diuretics and in children with nephritic syndrome [55].

The use of paracetamol is well tolerated in most cases but its use must be carefully monitored in children with hepatic impairment because of its hepatic metabolism and risk of hepatotoxicity in these patients [61, 62]. Caution is required when administering paracetamol to children who are malnourished, obese, or taking concurrent medications such as carbamazepine, isoniazid, phenobarbital, and other barbiturates [63].

Effective management of fever and pain requires tailoring antipyretics and analgesics to each patient's clinical profile, considering factors such as liver or kidney condition and concomitant medications, to maximize symptom relief and minimize adverse effects. Nonetheless, it remains essential to evaluate whether the infection is being managed effectively, as fever can be a key indicator of the treatment's progress and effectiveness.

## Recommendation No. 6: Multidisciplinary interventions

### Manage complex and/or complicated cases as a multidisciplinary team for more comprehensive and coordinated management of the pediatric patient.

The multidisciplinary approach to managing complex and complicated cases of SSTIs ensures improved clinical outcomes by providing a more comprehensive and coordinated care strategy for pediatric patients. By involving professionals from various disciplines such as dermatologists, infectious disease specialists, surgeons, and pediatricians, this approach facilitates thorough assessment and personalized treatment planning [64].

This collaborative effort allows for a thorough assessment of all aspects of the patient's condition, leading to an accurate diagnosis and effective treatment plans tailored to each patient's specific needs. Regular team meetings and effective communication are crucial components of this approach, allowing different perspectives and expertise to be integrated [65]. By coordinating care across multiple disciplines, this approach minimizes the risk of complications and ensures that all potential problems are addressed promptly. It also improves resource utilization by avoiding redundancy and optimizing the use of medical resources.

Overall, the management of complex and complicated SSTIs, as well as other infections, in a multidisciplinary team is a key strategy for providing pediatric patients with comprehensive, coordinated, high-quality care [66–68].

## Recommendation No. 7: Better assessment of appropriate dosages and administation intervals

### Carefully assess appropriate dosages during hospitalization and provide appropriate health education to parents/caregivers of patients regarding dosages and hourly intervals of administration of home-prescribed medications to improve therapeutic compliance

When assessing dosages, administration intervals and modalities during hospitalization, pharmacokinetic characteristics of antibiotics should be considered.

Medication nonadherence is a major problem in pediatric care and can lead to decreased treatment efficacy, inappropriate dosing, and increased risk of side effects [69–72]. Factors influencing adherence include parental understanding of the drug, and child behavioral and developmental issues [73].

Effective communication between parents and physicians is critical; positive interactions, appropriate education about the benefits of medications and their administration, and supportive advice from physicians are linked to better adherence [74, 75].

Errors in drug administration to children at home are common and can have serious consequences [76–79]. Contributing factors include prescribing practices, pharmacy dispensing errors, confusing dosing tools, multiple caregivers, and health literacy or language barriers [80, 81]. Physicians'prescribing practices can influence medication errors by simplifying or complicating the administration process for parents and caregivers. Incomplete or ambiguous information, such as details about route, frequency, duration, and indications, can affect the clarity of medication administration instructions [80].

Effective health education of parents about the dosages and administration intervals of home-prescribed medications is essential to improve therapeutic compliance and minimize medication errors. This, in turn, enhances the effectiveness of the treatment and reduces the risk of adverse effects or therapeutic failures.

Comprehensive education involves not only providing clear, written instructions but also engaging parents in verbal discussions to clarify any possible uncertainty. Clinicians should address potential questions, explain the importance of adhering to the specified intervals, and offer strategies for managing missed doses or modifying the schedule if necessary.

Investing in effective communication strategies is critical to achieving optimal treatment outcomes and promoting a collaborative approach to child care [82, 83].

## Recommendation No. 8: Appropriate communication with the patient's family

### Improve communication (active/passive) between doctor-caregiver-patient, both in terms of quality and time spent on it.

Clear, empathetic, and comprehensive communication increases trust and collaboration between physicians and caregivers, which is essential for successful pediatric care [84, 85].

By devoting adequate time and effort to explaining the patient's condition, treatment options, and care plan, physicians ensure that families fully understand the medical setting and their role in the care process. When families are well informed and involved, they are better equipped to manage patient care, recognize signs of complications, and provide the needed support. This collaborative relationship is crucial to optimize treatment adherence and compliance with prescribed therapies, to improve patient outcomes, and ensure that the provided care is in line with the family's expectations and needs [86, 87]. Therefore, investing in clear and thorough communication with the patient's family is critical to achieve positive and lasting therapeutic outcomes [84, 88].

Interpersonal and communication skills are essential for physicians, particularly in pediatrics, as these qualities have a significant impact on relationships with children and caregivers [89–92]. Furthermore, in this case, a relationship must be established among the physician, parent and child, thus making the process more complex and delicate.

An effective communication is based on informativeness (quality and quantity of health information), interpersonal sensitivity (physician's attention to the feelings and concerns of the parent and child), and partnership building (encouraging the parent and child to share their concerns and suggestions) [93].

Moreover, an effective time management is essential for physicians to gain the respect of patients and families, resulting in better outcomes. The use of closed questions to shorten consultations can prevent patients from fully expressing their concerns, potentially leading to misdiagnosis and adverse health outcomes; therefore, encouraging open communication improves the quality of consultations without prolonging their duration [94].

## Recommendation No. 9: Management of antibiotic therapy

### Make the best treatment choices based on comprehensive patient care, by choosing an antibiotic that reduces hospital stay, provides possible continuity for home care, and whose duration complies with the latest recommendations and guidelines

In clinical practice, optimal treatment choices require a comprehensive approach that balances the effective use of antibiotics with overall patient care. These choices should be tailored to the individual patient (organ failure, allergy to certain classes of antibiotics/drugs, need for hospitalization, possibility of early discharge and/or outpatient treatment, adherence to outpatient treatment) based on the characteristics of the available drugs (toxicity profile, availability of oral formulation, long-acting activity, and cost) in order to select the agent best suited to the needs of the individual patient, in line with the principles of precision medicine [95]. Prolonged hospitalization in children is associated with increased psychological stress, manifesting as anxiety, fear, withdrawal, and maladaptive responses, emphasizing the importance of early discharge and return to the habitual family routines [96, 97].

A holistic approach to therapy reduces nosocomial infections, bacterial resistance, and direct and indirect costs associated with prolonged hospitalizations, improving the quality of care overall.

To mitigate extended hospitalization, strategies such as Outpatient Parenteral Antimicrobial Therapy (OPAT) and transitioning to oral antibiotics have been employed. The OPAT strategy is often limited by logistical constraints, including the need to use an intravenous line daily and to closely follow the patient and monitor laboratory results [98]. Similarly, oral treatments often require multiple daily doses, which can challenge adherence in children, and may involve potential drug-drug interactions, side effects, and the need for regular laboratory monitoring.

These challenges often lead to delays in hospital discharge, underscoring the need for alternative therapeutic approaches. Long-acting lipoglycopeptides, such as dalbavancin, represent a promising solution, offering sustained antimicrobial coverage with one or two doses. This approach may facilitate hospital discharge by providing reliable systemic antimicrobial exposure for several weeks and ensuring completion of required treatment before or immediately after discharge [99].

Dalbavancin, now approved for treating ABSSSI in pediatric patients, can be administered in a single intravenous infusion, maintaining therapeutic levels in serum and tissues for over 7 days due to its long terminal half-life of 250 to 350 h (10–14 days) [100].

Although this drug does not yet appear to be widely used in pediatric clinical practice, as evidenced by the paucity of studies or case reports in the literature, dalbavancin offers a number of advantages: high efficacy, optimal safety profile, single administration, potential for early hospital discharge and reduced incidence of nosocomial infections, potent and prolonged bactericidal activity against Gram-positive bacteria (including MRSA), and no or reduced need for monitoring due to its low drug-drug interaction potential [101].

Similar to adults, dalbavancin may be used in selected pediatric patients with ABSSSI not eligible for oral antibiotics or OPAT programs. Compared with OPAT, long-acting antibiotics such as dalbavancin may require fewer contacts with the healthcare system and could reduce the inconvenience related to prolonged or repeated intravenous therapy by eliminating the need for central catheters, resulting in the prevention of line-related complications [102]. Additionally, long-acting antibiotics can mitigate the psychological burden associated with extended hospital stays by reducing the need for frequent healthcare-related interactions and interventions, facilitating an early return to normalcy.

The use of dalbavancin in pediatric settings may facilitate hospital discharge by switching from initial intravenous therapy, or even avoid unnecessary hospital admissions through its administration in outpatient clinic, as studies on the drug's use in the treatment of ABSSSI and its off-label application in the treatment of bone infections in children and adolescents have shown [103–105].

By incorporating this antibiotic into practice, pediatricians can improve clinical outcomes and quality of life for their young patients while reducing costs and complications associated with prolonged hospital stays [106–108].

## Recommendation No. 10: Post-discharge follow-up

### Strengthen the hospital-territory network, including cooperation with family pediatricians, to ensure continuity of care post-discharge.

Strengthening the hospital-territory network is essential to ensure continuity of care after discharge, improving patient outcomes and increasing the overall efficiency of the healthcare system. This entails enhancing communication with family pediatricians about post-discharge management, thus facilitating support and education of the families on the sustained benefits of long-acting therapies.

An integrated network of structured care and follow-up services can significantly reduce the risk of re-hospitalizations by ensuring that patients receive consistent and coordinated care even after leaving the hospital. This network facilitates better collaboration among healthcare providers, enabling seamless sharing of treatment choices and patient information. As a result, patients experience a better quality of life through continuity and consistency of care [109].

For the healthcare system, this integration results in increased efficiency by minimizing unnecessary hospital readmissions and optimizing the use of resources.

Moreover, continuity of care post-discharge is particularly beneficial for young patients, as it promotes increased therapeutic adherence by their parents. When parents are supported through a cohesive care network, they are more likely to follow prescribed treatments and attend follow-up appointments, thereby improving the health outcomes of their children.

Overall, an enhanced hospital-territory network not only benefits individual patients through improved health outcomes and quality of life, but also increases overall performance and health system sustainability [110].

## Conclusions

The management of SSTIs in pediatric patients presents complex clinical challenges that require a comprehensive and structured approach, especially in the presence of pain and fever. These infections vary in severity from mild cases to life-threatening conditions, requiring rapid diagnostic evaluation and individualized treatment strategies. Inadequate or delayed intervention can lead to disease progression, increased hospitalization rates, and increased burden on healthcare systems.

This opinion paper establishes a structured set of “golden rules” to guide physicians in optimizing the management of pediatric SSTIs. Early diagnosis, supported by targeted laboratory testing, is essential to distinguish bacterial infections from other diseases, thereby improving diagnostic accuracy and preventing unnecessary antibiotic use. Rational empiric therapy, guided by local epidemiology and individual patient risk factors, ensures effective initial treatment while minimizing the emergence of antimicrobial resistance. The timely switch to targeted therapy based on microbiological findings remains critical in refining antibiotic regimens, reducing treatment failure, and limiting adverse effects.

Beyond antibiotic stewardship, effective fever and pain management is fundamental in the care of pediatric SSTIs. A tailored approach, considering prior antipyretic/analgesic use and potential toxicity risks, enhances symptom control and prevents exacerbation of underlying infections. In more complex cases, a multidisciplinary team − including pediatricians, infectious disease specialists, microbiologists, and pharmacists − plays a crucial role in ensuring comprehensive patient care, optimizing treatment plans, and improving overall prognosis.

Moreover, ensuring appropriate dosing regimens and administration intervals is paramount, both during hospitalization and in home-based care. Structured communication between healthcare providers and caregivers fosters adherence to prescribed therapies, reduces medication errors, and improve treatment efficacy. Strengthening the transition from hospital to home care through close collaboration with family pediatricians and the integration of structured follow-up protocols further reduces the risk of relapse and improves long-term patient outcomes.

By implementing these targeted strategies, healthcare providers can significantly improve the quality of SSTI management in pediatric patients. This holistic approach not only ensures effective infection control and symptom relief, but also plays a key role in reducing antimicrobial resistance, optimizing healthcare resources, and ultimately improving the quality of life for affected children and their families. The adoption of these principles is essential for shaping a sustainable and efficient model of pediatric SSTI management, ensuring better outcomes both at an individual and public health level.

## Data Availability

Not applicable.

## Abbreviations

ABSSSI: Acute Bacterial Skin and Skin Structure Infections
ACE: Angiotensin-Converting Enzyme
AMR: Antimicrobial Resistance
AMS: Antimicrobial Stewardship
CNS: Central Nervous System
CRP: C-Reactive Protein
FDA: Food and Drug Administration
ICU: Intensive Care Unit
IV: Intravenous
MRSA: Methicillin-Resistant Staphylococcus aureus
NSAIDs: Non-Steroidal Anti-Inflammatory Drugs
OPAT: Outpatient Parenteral Antimicrobial Therapy
SSTIs: Skin and Soft Tissue Infections
TDM: Therapeutic Drug Monitoring
WHO: World Health Organization
